# Intracranial Atherosclerotic Burden and Cerebral Parenchymal Changes at 7T MRI in Patients With Transient Ischemic Attack or Ischemic Stroke

**DOI:** 10.3389/fneur.2021.637556

**Published:** 2021-05-06

**Authors:** Arjen Lindenholz, Jeroen de Bresser, Anja G. van der Kolk, H. Bart van der Worp, Theodoor D. Witkamp, Jeroen Hendrikse, Irene C. van der Schaaf

**Affiliations:** ^1^Department of Radiology, University Medical Center Utrecht, Utrecht, Netherlands; ^2^Department of Radiology, Leiden University Medical Center, Leiden, Netherlands; ^3^Netherlands Cancer Institute–Antoni van Leeuwenhoek Hospital, Amsterdam, Netherlands; ^4^Department of Neurology and Neurosurgery, University Medical Center Utrecht Brain Center, Utrecht University, Utrecht, Netherlands

**Keywords:** intracranial arteries, intracranial atherosclerosis, intracranial vessel wall MR imaging, small vessel disease, large vessel disease, white matter hyperintensity

## Abstract

The relevance of intracranial vessel wall lesions detected with MRI is not fully established. In this study (trial identification number: NTR2119; www.trialregister.nl), 7T MRI was used to investigate if a higher vessel wall lesion burden is associated with more cerebral parenchymal changes in patients with ischemic stroke or transient ischemic attack (TIA). MR images of 82 patients were assessed for the number of vessel wall lesions of the large intracranial arteries and for cerebral parenchymal changes, including the presence and number of cortical, small subcortical, and deep gray matter infarcts; lacunes of presumed vascular origin; cortical microinfarcts; and periventricular and deep white matter hyperintensities (WMHs). Regression analyses showed that a higher vessel wall lesion burden was associated with the presence of small subcortical infarcts, lacunes of presumed vascular origin, and deep gray matter infarcts (relative risk 1.18; 95% CI, 1.03–1.35) and presence of moderate-to-severe periventricular WMHs (1.21; 95% CI, 1.03–1.42), which are all manifestations of small vessel disease (SVD). The burden of enhancing vessel wall lesions was associated with the number of cortical microinfarcts only (1.48; 95% CI, 1.04–2.11). These results suggest an interrelationship between large vessel wall lesion burden and cerebral parenchymal manifestations often linked to SVD or, alternatively, that vascular changes occur in both large and small intracranial arteries simultaneously.

## Introduction

Intracranial atherosclerosis (ICAS) is an important cause of ischemic stroke (1). Historically, ICAS has been evaluated by measuring the presence of intracranial stenosis using lumenographic techniques or by detecting vessel wall calcifications that generally reflect a more advanced stage of ICAS (2–5). Over the last two decades, however, intracranial vessel wall MRI sequences have enabled *in vivo* visualization of the intracranial vessel wall itself (6–8). With these dedicated MRI sequences, both subtle (non-stenotic) and more advanced vessel wall pathology of the proximal cerebral large arteries can be assessed.

Vessel wall changes of the large intracranial arteries are frequently observed on intracranial vessel wall MR images, both in patients with cerebrovascular disease and in healthy elderly individuals, but the nature and clinical relevance of these changes have not been fully established (9–12). *Ex vivo* studies applying intracranial vessel wall MRI to postmortem samples suggest that these changes represent vessel wall disease at an early stage to halfway on the developmental timeline of ICAS (13–18). At the end of this timeline, plaque disruption, thrombus formation, and large vessel stenosis or occlusion can lead to transient ischemic attack (TIA) or ischemic stroke. The parenchymal consequences of vessel wall changes presumably reflecting less advanced ICAS, however, are less clear, hampering interpretation of these changes in clinical practice.

ICAS in the smaller arteries, arterioles, and capillaries—while difficult to visualize using vessel wall MRI due to their small caliber—or emboli disrupted from atherosclerotic plaques located in larger parent arteries may lead to (small) subcortical infarcts, deep gray matter infarcts, lacunes of presumed vascular origin, cortical microinfarcts, or white matter hyperintensities (WMHs) (19, 20). Often, these cerebrovascular changes of the small vessels and their sequelae are categorized as a separate disease entity [cerebral small vessel disease (SVD)] compared with large artery stroke, although both small and large cerebral arteries comprise one vascular bed that is physiologically connected, suggesting that pathology in one “type” of vessel bed will inevitably affect the other. Recent studies have shown evidence for this connection: for example, large (intracranial) artery disease may result in endothelial damage and blood–brain barrier damage of the smaller arteries by the release of inflammatory molecules and enzymes, while alternatively, atherosclerotic plaques located in the larger intracranial arteries may obstruct orifices of smaller branching arteries resulting in small vessel pathology (21, 22). Nevertheless, the exact role and direction of effect—cerebrovascular changes of the small arteries by large artery pathology, vice versa, or bilateral—remains to be elucidated (22).

In this study, we hypothesize that the burden of intracranial vessel wall lesions of the larger intracranial arteries might be associated with a variety of cerebral parenchymal changes, including large and small infarcts and WMHs. The purpose of this study was therefore to investigate whether a greater burden of intracranial vessel wall lesions assessed using 7-tesla (T) vessel wall MRI is associated with more cerebral parenchymal changes.

## Materials and Methods

### Study Population

**Figure 1:**
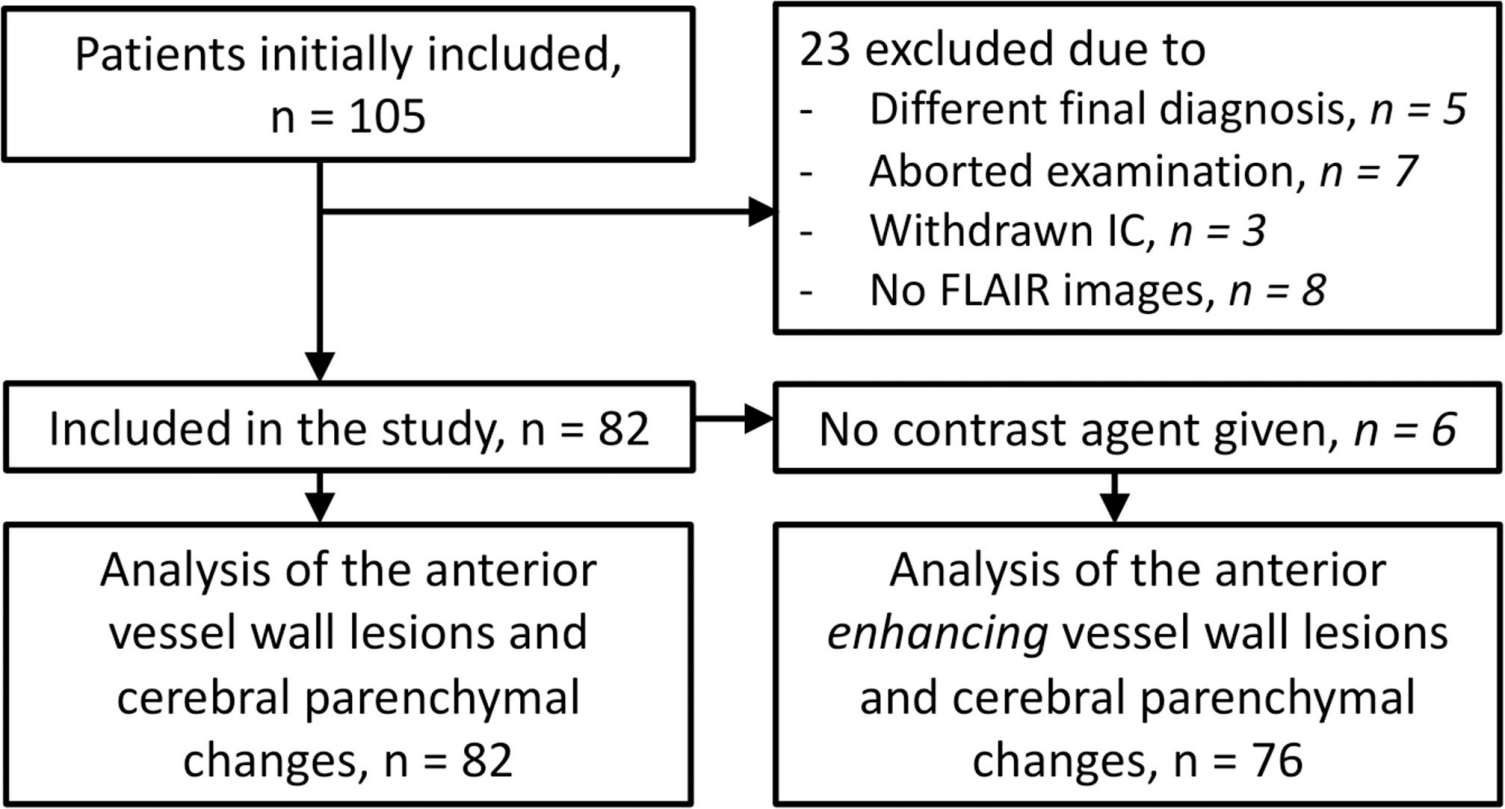
Flowchart showing the number of participants who were included for analyses. IC, informed consent.

Between December 2009 and May 2018, patients presenting at our University hospital with TIA or ischemic stroke of the anterior circulation were eligible for inclusion in the prospective Intracranial Vessel wall Imaging (IVI) study (NTR2119, www.trialregister.nl). The main inclusion criteria were age 18 years or older and ability to undergo a 7T MRI examination within 3 months after the ischemic event. The main exclusion criteria were ischemic stroke or TIA secondary to a surgical or interventional procedure and any contraindication to 7T MR imaging or to gadolinium-containing contrast agents. A flowchart of the study inclusion is shown in Figure 1. The full eligibility criteria and other details of the study have been reported before (6, 23). The study was approved by the medical ethics committee of the University Medical Center Utrecht according to the guidelines of the Declaration of Helsinki of 1975, and all patients provided written informed consent. Baseline characteristics including cardiovascular risk factors and characteristics of the ischemic event [final diagnosis and subtype classified by the Trial of Org 10172 in Acute Stroke Treatment (TOAST) criteria] were recorded (24).

### Imaging Protocol

Imaging was performed on a 7T whole-body system (Philips Healthcare, Best, The Netherlands) with either a 16-channel (*n* = 35) or 32-channel (*n* = 47) receive coil and a volume transmit-receive coil for transmission (Quad TR; Nova Medical, Wilmington, MA, USA). The protocol included a pre- and post-contrast T_1_-weighted intracranial vessel wall sequence [magnetization-prepared inversion recovery turbo spin-echo (MPIR-TSE)], time-of-flight MR angiography (TOF-MRA), diffusion-weighted imaging (DWI), and fluid-attenuated inversion recovery (FLAIR) imaging. The post-contrast MPIR-TSE sequence was acquired 5 min after the administration of 0.1 ml/kg of a gadolinium-containing contrast agent (gadobutrol, Gadovist 1.0 mmol/ml, Bayer Schering Pharma, Newbury, UK). The TOF-MRA images were used for anatomical verification of the large intracranial vessels seen on the MPIR-TSE images. The following acquisition parameters were used for the MPIR-TSE sequence: field of view (FOV) 220 × 180 × 13 mm^3^ (*n* = 40), which was extended during the study period to 250 × 250 × 190 mm^3^ (*n* = 42) (23), acquired spatial resolution 0.8 × 0.8 × 0.8 mm^3^, reconstructed spatial resolution 0.5 × 0.5 × 0.5 mm^3^, repetition time (TR) 3,952 ms, echo time (TE) 37 ms, inversion time (TI) 1,375 ms, and acquisition time 10 min 40 s. In case of the smaller FOV sequence, the FOV was placed in such a way that all large intracranial arteries of the anterior circulation were within the imaging plane, which included the anterior cerebral arteries (ACAs; A1 and A2 segments), middle cerebral arteries (MCAs; M1 and M2 segments), and the distal parts of the internal (intracranial) carotid arteries (ICAs; clinoid, supra-clinoid, and terminal segments). Acquisition parameters for the other sequences were as follows: for the TOF-MRA, FOV 190 × 190 × 102 mm^3^, acquired spatial resolution 0.4 × 0.5 × 0.6 mm^3^, reconstructed spatial resolution 0.4 × 0.4 × 0.3 mm^3^, TR 21 ms, TE 2.3 ms, and acquisition time 9 min 18 s; for the DWI, FOV 220 × 220 × 123 mm^3^, acquired and reconstructed spatial resolution 1.5 × 1.5 × 1.5 mm^3^, TR 17,659 ms, TE 57 ms, and acquisition time 6 min 10 s; and for the FLAIR sequence, FOV 250 × 250 × 190 mm^3^, acquired spatial resolution 0.8 × 0.8 × 0.4 mm^3^, reconstructed spatial resolution 0.5 × 0.5 × 0.5 mm^3^, TR 8,000 ms, TE 300 ms, TI 2,200 ms, and acquisition time 10 min 48 s [for more details, see Lindenholz et al. (11)].

### Vessel Wall Assessment

The MR vessel wall images were scored by an expert in reading intracranial vessel wall images (AK, 9 years of experience). The reader was blinded for patient details and clinical information. Interrater reproducibility measures for the assessment of these vessel wall images have been published before [intraclass correlation coefficient, 0.93; 95% confidence interval (CI); 0.90–0.96; Dice similarity coefficient (DSC), 0.81] (25). For the current study, intracranial vessels of the anterior circulation were assessed, including the ACA (A1 and A2 segments), MCA (M1 and M2 segments), and the ICA (clinoid, supra-clinoid, and terminal segments). A vessel wall lesion was defined as a visually judged focal or diffuse vessel wall thickening of >50% compared with the neighboring vessel wall (or contralateral vessel wall in case of diffuse thickening), with or without contrast enhancement (11). Contrast enhancement was assessed by comparing pre- and post-contrast images including co-registered subtraction images using the Elastix toolbox in MeVisLab (version 2.7, MeVis Medical Solutions, Bremen, Germany) (26). Contrast enhancement of a vessel wall lesion was defined as lesion hyperintensity approximating the signal intensity of the pituitary stalk on the post-contrast MR images on at least two consecutive images using the pre-contrast MR images as a reference and the coregistered subtraction images as confirmation. Contrast-enhancing foci at the location where the ICA pierces the dura mater were classified as vasa vasorum and were not considered vessel wall enhancement. The intracranial vessel wall lesion burden was used as a continuous variable for the primary analysis and was defined as the total number of vessel wall lesions for each patient. The total number of enhancing vessel wall lesions was used as a continuous variable in the secondary analysis.

### Parenchyma Assessment

The DWI and FLAIR images and T_1_-weighted MPIR-TSE images were assessed for anterior circulation parenchymal changes by an expert neuroradiologist (TDW, 30 years of experience). The reader was blinded for patient details, clinical information, and vessel wall lesion assessment. The following cerebral parenchymal changes were scored: cortical infarcts and (recent) small subcortical infarcts, deep gray matter infarcts and lacunes of presumed vascular origin, cortical microinfarcts, and periventricular and deep WMHs. A recent position paper has judged all of these MR findings except for cortical infarcts (and possibly cortical microinfarcts) to be related to SVD (27).

Cortical infarcts were defined as hyperintense lesions on FLAIR imaging located in the cerebral cortex and >5 mm in diameter, with or without associated tissue loss or extension into the deep white matter. When a cortical infarct was multifocal or extending into the white matter, but with similar presumed origin, the infarct was scored as a single cortical infarct. Small subcortical infarcts and lacunes of presumed vascular origin were defined as hyperintense lesions of, respectively, 15–20 and 3–15 mm in diameter on FLAIR imaging with or without a hypointense center or cavity and local tissue loss, classified and scored according to the STRIVE criteria (27). Deep gray matter infarcts were defined as hyperintense lesions on FLAIR imaging located in the basal ganglia or thalamus >3 mm in diameter (not specifically defined in the STRIVE criteria but derived from the position paper) (27). Cortical microinfarcts were defined as hyperintense lesions on FLAIR imaging in the cerebral cortex smaller than 5 mm in size and scored according to a recent consensus paper (20). The periventricular and deep WMHs on FLAIR imaging were also defined according to the STRIVE criteria and were scored with the Fazekas four-point scale for periventricular (0 = absent WMH lesions, 1 = “caps” or pencil-thin lining, 2 = smooth “halo,” and 3 = irregular hyperintensities extending into the deep white matter) and for deep WMHs (0 = absence or a single punctate WMH lesion, 1 = multiple punctate lesions, 2 = beginning confluency of lesions, and 3 = large confluent lesions) (27, 28). The presence of any cerebral infarct in the flow territory of the anterior circulation and the WMH score (dichotomized into high, Fazekas 2 or 3; and low, Fazekas 0 or 1) were used as primary outcomes in the analyses. The number of cerebral infarcts was used as outcomes for the secondary analysis.

### Statistical Analysis

Descriptive baseline statistics are presented as proportions or means. To estimate associations between intracranial vessel wall lesion burden and presence or number of cerebral parenchymal changes, appropriate regression analyses for modeling count data were performed. Age and sex were included as covariates. In the primary analyses, the association between the number of intracranial vessel wall lesions (as continuous and independent variable) and the presence of any scored cerebral parenchymal change (dichotomized outcome as dependent variable) were individually and all together investigated with a negative log-binomial regression model. A composite variable was composed of the infarcts that are often considered to be manifestations of cerebral SVD and included small subcortical and deep gray matter infarcts and lacunes of presumed vascular origin (27). In the secondary analyses, the associations between the total number of intracranial vessel wall lesions (independent variable) and the total number of infarcts (count data; classified by infarct type, dependent variable) were investigated with a negative log-binomial regression model for the number of infarcts. To assess the association between the number of enhancing intracranial vessel wall lesions (independent variable) and presence and number of the scored cerebral parenchymal changes as dependent variables, we used the same methods as in the primary and secondary analyses. Negative log-binomial regression generally provides rate ratios, which are interpretable as relative risks. Therefore, associations are presented in more commonly used relative risks. For all analyses, 95% CIs are given. A two-sided *p* < 0.05 was considered statistically significant. Statistical analyses were performed using SPSS version 21.0 (IBM SPSS Statistics, IBM Corp., Armonk, NY, USA).

## Results

A total of 105 patients were included in the IVI study. Twenty-three patients were excluded because of reasons listed in Figure 1, leaving a total of 82 patients for the current study. Baseline characteristics of the study participants are shown in Table 1.

**Table 1:** Baseline characteristics.

**Patient characteristics (*n* = 82)**	
Mean age in years (range)	61 (27–85)
Men	49 (60%)
Body mass index (BMI, kg/m^2^), mean (range)	26 (18–35)
**Diagnosis**	
Ischemic stroke	55 (67%)
Transient ischemic attack	22 (27%)
Transient monocular visual loss of vascular origin	5 (6%)
Hypertension	40 (49%)
Hyperlipidemia	39 (48%)
Diabetes mellitus	10 (12%)
Peripheral artery disease	0 (0%)
Current smoker	26 (32%)
Former smoker	27 (33%)
Atrial fibrillation	10 (12%)
Angina pectoris	4 (5%)
Myocardial infarction	5 (6%)
Average days from symptom onset to MRI in days ± SD	23 ± 34
Patients imaged without contrast agent	6 (7%)
**TOAST-criteria**	
Large artery atherosclerosis	44 (54%)
Cardio embolism	17 (21%)
Small-vessel occlusion	5 (6%)
Other determined etiology	5 (6%)
Undetermined	11 (13%)

*Patient characteristics for the studied population*.

### Distribution of Intracranial Vessel Wall Lesions and Cerebral Parenchymal Changes

Sixty-two patients (75%) had a total of 193 vessel wall lesions and 35 patients (43%) a total of 81 enhancing vessel wall lesions. The total number of (enhancing) vessel wall lesions per arterial segment is shown in Supplementary Table 1.

Sixty-eight patients (83%) had at least one cerebral parenchymal vascular lesion. Fifty-five patients (67%) had a total of 122 cortical infarcts. One patient (1%) had a small subcortical infarct of <20 mm, 23 patients (28%) had a total of 54 lacunes of presumed vascular origin, 15 patients (18%) had a total of 21 subcortical deep gray matter infarcts, and 16 patients (20%) had a total of 43 cortical microinfarcts. For the periventricular WMHs and for the deep WMHs, 24 patients (29%) and 25 patients (30%) had a Fazekas score of 2 and 3, respectively. All details regarding the detected cerebral parenchymal changes are shown in Supplementary Table 2.

### Intracranial Vessel Wall Lesion Burden and Cerebral Parenchymal Changes

**Figure 2:**
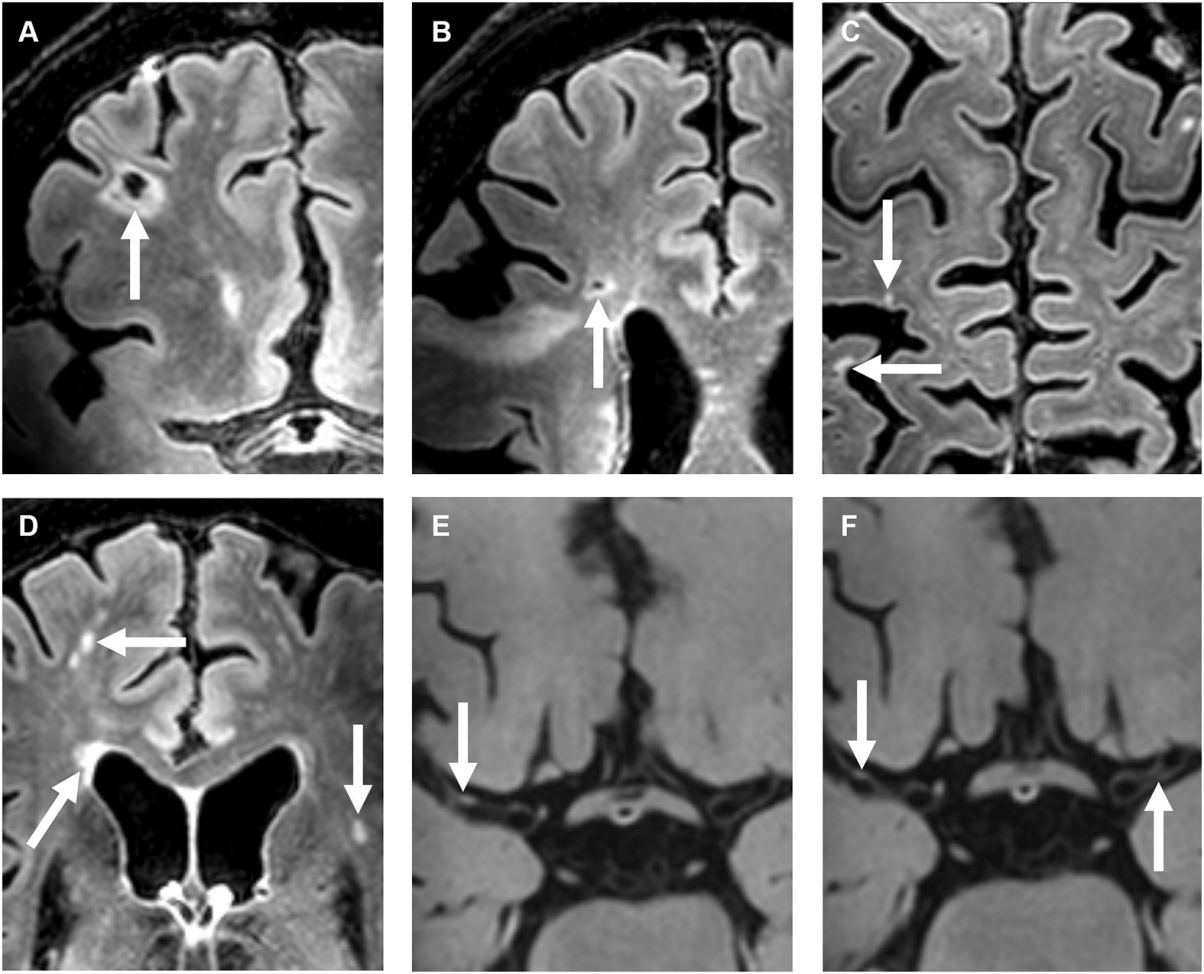
A 72-year-old female with a right-sided ischemic stroke; imaging 25 days after symptom onset. **(A–D)** Precontrast 7T transverse FLAIR images and **(E–H)** post-contrast 7T transverse T_1_-weighted MPIR-TSE vessel wall images. Lacunar infarcts are located in the right frontal [arrow in **(A)**] and right frontotemporal area [arrow in **(B)**]. Multiple cortical microinfarcts are also seen [arrows in **(C)**], as well as periventricular and deep white matter hyperintensities [arrows in **(D)**]. Note also the extensive enlarged periventricular spaces in this patient [not scored; arrows in **(E,F)**]. Vessel wall lesions are seen in the left and right middle cerebral arteries (M1 segments) [arrows in **(G,H)**].

Unadjusted and adjusted relative risks for the association between intracranial vessel wall lesion burden and the presence of cerebral parenchymal changes are shown as primary analyses in Table 2. In our study population, vessel wall lesion burden was not associated with the presence of small cortical infarcts (adjusted relative risk, 1.07; 95% CI, 0.98–1.17). Neither was an association found between vessel wall lesion burden and presence of lacunes of presumed vascular origin (adjusted relative risk, 1.13; 95% CI, 0.95–1.34), deep gray matter infarcts (adjusted relative risk 1.25; 95% CI, 0.98–1.60), and cortical microinfarcts (adjusted relative risk 1.07; 95% CI, 0.85–1.36). When all subcortical infarcts were combined into one composite variable composing the infarcts often considered to be a manifestation of SVD, an association was found between vessel wall lesion burden and presence of an infarct (adjusted relative risk, 1.18; 95% CI, 1.03–1.35). In addition, the presence of moderately to severe (Fazekas 2–3) periventricular WMHs was associated with vessel wall lesion burden (adjusted relative risk, 1.21; 95% CI, 1.02–1.42). No association was found between deep WMHs and vessel wall lesion burden. An example of the presence of vessel wall lesions and a high WMH score is shown in Figure 2.

**Table 2:** Associations between the presence of parenchymal changes and the intracranial vessel wall lesion burden.

**Outcome as present/absent**	**Unadjusted relative risk (±95% CI)**	***p*-value**	**Adjusted relative risk (±95% CI)**	***p*-value**
**Infarcts**				
Any anterior circulation infarct	1.05 (0.99–1.12)	0.086	1.05 (0.98–1.12)	0.151
Cortical infarcts	1.06 (0.98–1.15)	0.151	1.07 (0.98–1.17)	0.129
Infarcts often caused by SVD	**1.23 (1.09–1.39)**	**0.001**	**1.18 (1.03–1.35)**	**0.017**
Small subcortical infarcts	NA		NA	NA
Lacunes of presumed vascular origin	**1.20 (1.04–1.40)**	**0.016**	1.13 (0.95–1.34)	0.174
Deep gray matter infarcts	**1.29 (1.04–1.60)**	**0.019**	1.25 (0.98–1.60)	0.069
Cortical microinfarcts	1.14 (0.93–1.38)	0.212	1.07 (0.85–1.36)	0.565
**White matter hyperintensities (Fazekas grade)**				
Periventricular (0/1 vs. 2/3)	**1.34 (1.16–1.55)**	**<0.001**	**1.21 (1.02–1.42)**	**0.026**
Deep (0/1 vs. 2/3)	**1.22 (1.05–1.41)**	**0.008**	1.09 (0.89–1.33)	0.417

*Included for analysis, n = 82. The unadjusted and adjusted (for age and sex) relative risks including their 95% confidence interval (CI) for the presence of cerebral parenchymal changes with the total number of anterior vessel wall lesions as included variable. A p < 0.05 was considered to indicate a statistically significant difference shown as bold text. A log-binomial regression model was used with a robust variance estimator. White matter hyperintensity Fazekas grade is described as follows: 0 = absence or single punctate white matter hyperintensity, 1 = “caps” or pencil-thin lining or multiple punctate lesions, 2 = smooth “halo” or beginning confluency of lesions, and 3 = large confluent lesions or irregular hyperintensities extending into the deep white matter (28)*.

*CMI, cortical microinfarct; NA, not applicable (only 1 count); SVD, small vessel disease*.

In the secondary analyses, we did not find an association between intracranial vessel wall lesion burden and the number of cerebral parenchymal changes of any type (Table 3). In the subanalyses, an association was found between the enhancing vessel wall lesion burden and the number of cortical microinfarcts (relative risk, 1.48; 95% CI, 1.04–2.11), but no other association was found between the enhancing vessel wall lesion burden and either presence (Table 4) or number (Table 5) of cerebral parenchymal changes.

**Table 3:** Associations between the number of parenchymal changes and the intracranial vessel wall lesion burden.

**Outcome as count**	**Unadjusted relative risk (±95% CI)**	***p*-value**	**Adjusted relative risk (±95% CI)**	***p*-value**
**Infarcts**				
Any anterior circulation infarct	**1.15 (1.03–1.29)**	**0.016**	1.08 (0.96–1.21)	0.189
Cortical infarcts	1.12 (0.98–1.28)	0.102	1.08 (0.94–1.24)	0.290
Infarcts often caused by SVD	1.12 (0.97–1.31)	0.134	1.05 (0.90–1.23)	0.531
Small subcortical infarcts	NA	NA	NA	NA
Lacunes of presumed vascular origin	1.09 (0.90–1.31)	0.370	1.00 (0.79–1.26)	0.981
Deep gray matter infarcts	1.21 (0.91–1.61)	0.190	1.15 (0.84–1.58)	0.396
Cortical microinfarcts	1.23 (0.95–1.60)	0.111	1.16 (0.86–1.56)	0.333
**White matter hyperintensities (Fazekas grade)**				
Periventricular (0, 1, 2, or 3)	NA	NA	NA	NA
Deep (0, 1, 2, or 3)	NA	NA	NA	NA

*Included for analysis, n = 82. The unadjusted and adjusted (for age and sex) relative risks including their 95% confidence interval (CI) for the number of cerebral parenchymal changes with the total number of anterior vessel wall lesions as included variable. A p < 0.05 was considered to indicate a statistically significant difference shown as bold text. For continuous count data as outcome variable, a log-binomial regression model was used with a robust variance estimator. For ordinal data as outcome variable, an ordinal regression model was used. Ordinal Fazekas grade is described as follows: 0 = absence or single punctate white matter hyperintensity, 1 = “caps” or pencil-thin lining or multiple punctate lesions, 2 = smooth “halo” or beginning confluency of lesions, and 3 = large confluent lesions or irregular hyperintensities extending into the deep white matter (28)*.

*CMI, cortical microinfarct; NA, not applicable (groups too small for reliable statistical analyses); SVD, small vessel disease*.

**Table 4:** Associations between the presence of parenchymal changes and the enhancing intracranial vessel wall lesion burden.

**Outcome as present/absent**	**Unadjusted relative risk (±95% CI)**	***p*-value**	**Adjusted relative risk (±95% CI)**	***p*-value**
**Infarcts**				
Any anterior circulation infarct	1.00 (0.92–1.09)	0.957	0.99 (0.90–1.08)	0.824
Cortical infarcts	1.04 (0.95–1.14)	0.394	1.05 (0.94–1.16)	0.387
Infarcts often caused by SVD	1.06 (0.90–1.26)	0.498	1.00 (0.83–1.22)	0.975
Small subcortical infarcts	NA	NA	NA	NA
Lacunes of presumed vascular origin	1.07 (0.87–1.32)	0.537	1.00 (0.78–1.28)	0.986
Deep gray matter infarcts	0.90 (0.65–1.24)	0.508	0.83 (0.61–1.14)	0.256
Cortical microinfarcts	1.24 (0.95–1.60)	0.112	1.18 (0.93–1.49)	0.187
**White matter hyperintensities (Fazekas grade)**				
Periventricular (0/1 vs. 2/3)	**1.19 (0.98–1.45)**	**0.077**	1.02 (0.81–1.29)	0.858
Deep (0/1 vs. 2/3)	1.12 (0.92–1.36)	0.273	0.99 (0.80–1.24)	0.948

*Included for analysis, n = 76. Six of 82 patients did not receive contrast agent. The unadjusted and adjusted (for age and sex) relative risks including their 95% confidence interval (CI) for the presence of cerebral parenchymal changes with the total number of anterior enhancing vessel wall lesions as included variable. A p < 0.05 was considered to indicate a statistically significant difference shown as bold text. A log-binomial regression model was used with a robust variance estimator. White matter hyperintensity Fazekas grade is described as follows: 0 = absence or single punctate white matter hyperintensity, 1 = “caps” or pencil-thin lining or multiple punctate lesions, 2 = smooth “halo” or beginning confluency of lesions, and 3 = large confluent lesions or irregular hyperintensities extending into the deep white matter (28)*.

*CMI, cortical microinfarct; NA, not applicable (only 1 count); SVD, small vessel disease*.

**Table 5:** Associations between the number of parenchymal changes and the enhancing intracranial vessel wall lesion burden.

**Outcome as count**	**Unadjusted relative risk (±95% CI)**	***p*-value**	**Adjusted relative risk (±95% CI)**	***p*-value**
**Infarcts**				
Any anterior circulation infarct	1.17 (0.97–1.40)	0.096	1.10 (0.93–1.29)	0.269
Cortical infarcts	1.04 (0.93–1.15)	0.539	1.01 (0.91–1.12)	0.830
Infarcts often caused by SVD	1.01 (0.82–1.23)	0.918	0.97 (0.77–1.21)	0.773
Small subcortical infarcts	NA	NA	NA	NA
Lacunes of presumed vascular origin	1.06 (0.84–1.33)	0.636	1.00 (0.77–1.31)	0.984
Deep gray matter infarcts	0.82 (0.57–1.18)	0.278	0.75 (0.53–1.06)	0.102
Cortical microinfarcts	**1.59 (1.08–2.34)**	**0.019**	**1.48 (1.04–2.11)**	**0.032**
**White matter hyperintensities (Fazekas grade)**				
Periventricular (0, 1, 2, or 3)	NA	NA	NA	NA
Deep (0, 1, 2, or 3)	NA	NA	NA	NA

*Included for analysis, n = 76. Six of 82 patients did not receive contrast agent. The unadjusted and adjusted (for age and sex) relative risks including their 95% confidence interval (CI) for the number of cerebral parenchymal changes with the total number of anterior enhancing vessel wall lesions as included variable. A p < 0.05 was considered a statistically significant difference shown as bold text. For continuous count data as outcome variable, a log-binomial regression model was used with a robust variance estimator. For ordinal data as outcome variable, an ordinal regression model was used. Ordinal score is described as follows: 0 = absence or single punctate white matter hyperintensity, 1 = “caps” or pencil-thin lining or multiple punctate lesions, 2 = smooth “halo” or beginning confluency of lesions, and 3 = large confluent lesions or irregular hyperintensities extending into the deep white matter (28)*.

*CMI, cortical microinfarct; NA, not applicable (groups too small for reliable statistical analyses); SVD, small vessel disease*.

## Discussion

In the current study, an increased vessel wall lesion burden was associated with the presence of small subcortical infarcts, lacunes of presumed vascular origin, or deep gray matter infarcts, and with the presence of moderate-to-severe periventricular WMHs. We did not find an association between intracranial vessel wall lesion burden and presence and number of large cortical infarcts and presence and number of infarcts when classified by type. In addition, the enhancing vessel wall lesion burden was only associated with the number of cortical microinfarcts.

Several of the cerebral parenchymal changes that we assessed in our study have been considered manifestations of SVD (27). SVD is generally defined by cerebral parenchymal manifestations, as SVD itself is difficult to quantify (19, 29–31). By contrast, large artery disease can be assessed directly using either lumenography techniques (stenosis) or intracranial vessel wall imaging (vessel wall lesions) (9). Many studies focus on either large vessel disease or SVD, and ongoing effort has been committed to find differences or similarities between cerebral large artery disease and SVD (32–39). Despite anatomical and pathophysiological differences between large and small intracranial vessels, the intracranial vasculature is interrelated as one vascular network. SVD has recently been described as a dynamic whole-brain disease that in some cases may share a similar etiology with large vessel disease. In this proposed “parent artery atheroma theory,” atherosclerotic plaques in the large cerebral arteries (e.g., MCA) cause an occlusion at the origin of smaller branching arteries, subsequently leading to cerebral parenchymal changes reflecting SVD (21, 39). The findings in our study can support this theory, as we found an association between the presence of infarcts often related to SVD and the intracranial vessel wall lesion burden (Table 2). Specific microbiota, proteases, and immunoglobulins from atherosclerotic plaques can cause a cascade that harms the vessel wall endothelium and the blood–brain barrier and consequently could lead to SVD (22). This may be an alternative explanation of our findings that the co-occurrence of intracranial vessel wall lesions and parenchymal manifestations of SVD may suggest concomitant changes in both large and small intracranial vasculature.

It remains uncertain why an association was found between the enhancing vessel wall lesion burden and number of cortical microinfarcts, but not with the presence of cortical microinfarcts nor with the presence or number of any other type of infarct. This is most likely related to insufficient statistical power. Increasing evidence, including pooled data, shows that vessel wall lesion enhancement may be associated with plaque vulnerability and consequently with higher risk of ischemic events (40–42). Histopathological validation and especially follow-up studies with a larger sample size are necessary to investigate whether this applies to our study population.

Several etiologies have been described that attempt to explain different categories of WMHs, yet it remains unclear if there indeed exist distinct mechanisms for periventricular and deep WMH development and if these differences are of clinical importance (19, 43, 44). The presence of both periventricular and deep WMHs may be a result of underlying vascular changes (and subsequent loss of vascular integrity) of both large and SVD (19). Nevertheless, different associations between periventricular and deep WMHs were found in our study, and definite explanations for these discrepancies have yet to be further investigated.

In contrast to previous studies investigating ICAS as a risk factor for (large artery) ischemic stroke, in our study, no association was found between intracranial vessel wall lesion burden and presence and number of (large) cortical infarcts. This may be due to the setup of the IVI study, for which specifically ischemic stroke and TIA patients were selected, not limited to large-artery atherosclerosis according to the TOAST criteria, and without inclusion of healthy volunteers. Large numbers of patients are needed to detect small differences in this type of study population. Another explanation (especially for the number of cortical infarcts) may be the difference in definition of ICAS. Previous studies often used intracranial stenosis as a proxy for ICAS, but this represents an advanced state of ICAS (45, 46). In early-to-moderate stages of ICAS, vessel wall changes may be its only (early) sign; due to vascular remodeling, luminal narrowing often occurs in advanced stages of the disease (47).

The strength of this study is that data have been derived from a relatively large number of patients who received 7T MR vessel wall imaging; high-resolution 7T MRI has been shown superior to 3T MRI in visualizing the intracranial vessel wall because of its high contrast-to-noise ratio and has a better detection rate of especially small symptomatic and asymptomatic cerebral parenchymal changes (12, 48). Still, for analyzing all associations in this study, the absolute number of included patients is limited, and the inherently induced selection bias due to the design of the study may have affected the analyzed associations underestimating the true association due to reversed causality. Also, the smaller size of the subgroup analysis may have accounted for the lack of an association, which also applies for the analyses with the enhancing vessel wall lesion burden as included variable. Although we detected several trends in our analyses, most of them could not be confirmed with statistical significance. We think that this can for the most part be explained by statistical power constraints. When periventricular and deep WMHs share the same etiology, the same reason may account for the observed difference in associations between the presence of periventricular vs. deep WMHs and vessel wall lesion burden. Overall, there was an overrepresentation of patients with a Fazekas score of 1 in both periventricular and deep WMHs, implicating relatively small subgroups of patients with a different Fazekas score. Therefore, (ordinal) regression analyses could not be reliably performed among the individual Fazekas scores. Also, we based our results on multivariable models and tested seven variables of cerebral parenchymal changes with the presence of (enhancing) vessel wall lesions. The Bonferroni correction for multiple testing would mean that our *p*-value of 0.05 should be divided by the nine outcome variables = 0.009, which indicates statistical significance. That approach would leave no association between (enhancing) vessel wall lesions and cerebral parenchymal changes. Furthermore, several other cerebral parenchymal changes have been recognized to reflect (chronic) vascular damage due to SVD, such as cerebral microbleeds, which were not assessed in this study (27). Ideally, the inclusion of a larger group of patients could improve the statistical reliability and enable inclusion of more potential confounders or effect modifiers, while assessment of these other cerebral parenchymal changes may contribute to a broader understanding of the potential associations between intracranial vessel wall lesion burden reflecting underlying large artery disease and the cerebral sequelae associated with SVD. Another limitation of assessing intracranial vessel wall lesion burden is the absence of a gold standard method to assess ICAS lesions found on MRI. However, this is a limitation of all studies on intracranial vessel wall MRI, and several post-mortem *ex vivo* imaging correlation studies have been performed, tentatively increasing the confidence that detected vessel wall lesions represent true ICAS (15, 18, 49). Yet the cutoff point between confirmed early-stage atherosclerotic lesions and non-pathological or natural variations of vessel wall thickness may be difficult to define.

## Conclusions

Within this relatively small sample size, intracranial vessel wall lesion burden was associated with the presence of periventricular WMHs and the presence of any type of infarct often linked to SVD, while no association was found with (large) cortical infarcts. The co-occurrence of intracranial vessel wall lesions and parenchymal manifestations generally attributed to SVD tentatively suggests that either vessel wall lesions of the large intracranial parent arteries eventually result in cerebral parenchymal manifestations of SVD, e.g., by occluding the orifices of smaller branching arteries, or vascular changes occur in both large and small intracranial arteries simultaneously. In the future, studies with larger sample sizes are required to confirm these findings.

## Data Availability Statement

The original contributions generated for the study are included in the article/Supplementary Material, further inquiries can be directed to the corresponding author/s.

## Ethics Statement

The studies involving human participants were reviewed and approved by the medical ethics committee of the University Medical Center Utrecht according to the guidelines of the Declaration of Helsinki of 1975. The patients/participants provided their written informed consent to participate in this study.

## Author Contributions

AL: study design, literature search, figures, data collection, data analyses, data interpretation, and writing. JdB: study design, literature search, data interpretation, and critical review of the manuscript. AvK: study design, data collection, data interpretation, and critical review of the manuscript. HvW: data interpretation and critical review of the manuscript. TW: MRI analyses. JH: study design and critical review of the manuscript. IS: study design, data interpretation, and critical review of the manuscript. All authors contributed to the article and approved the submitted version.

## Conflict of Interest

The authors declare that the research was conducted in the absence of any commercial or financial relationships that could be construed as a potential conflict of interest.

## Abbreviations

ACA: anterior cerebral artery
CI: confidence interval
FLAIR: fluid-attenuated inversion recovery
ICA: internal carotid artery
ICAS: intracranial atherosclerosis
MCA: middle cerebral artery
MPIR-TSE: magnetization-prepared inversion-recovery turbo spin echo
SVD: small vessel disease
T: tesla
TIA: transient ischemic attack
TOAST: Trial of Org 10172 in Acute Stroke Treatment
WMH: white matter hyperintensity.
