# Supplementary material for: Intracranial Atherosclerotic Burden and Cerebral Parenchymal Changes at 7T MRI in Patients With Transient Ischemic Attack or Ischemic Stroke
Source: Front Neurol. 2021 May 6;12:637556. doi: 10.3389/fneur.2021.637556 (PMC8134532; doi:10.3389/fneur.2021.637556)
Supplement: Supplementary file 1 [file Table_1.DOCX]

**ONLINE SUPPLEMENTAL MATERIAL**

**Online Supplemental Table 1.** The total number of (enhancing) vessel wall lesions of the anterior circulation, specified by arterial segment.

| ***Location*** | *Left (mean)* | *Left enhancing (mean)* | *Right (mean)* | *Right enhancing (mean)* | *Total (mean)* | *Total enhancing (mean)* |
| --- | --- | --- | --- | --- | --- | --- |
| Anterior cerebral artery |  |  |  |  |  |  |
| *A1 segment* | 5 (6%)^B^ | 0 (0%)^E^ | 9 (11%)^A^ | 1 (1%)^D^ | 14 | 1 |
| *A2 segment* | 2 (3%)^C^ | 0 (0%)^D^ | 7 (9%)^B^ | 3 (4%)^D^ | 9 | 3 |
| Middle cerebral artery |  |  |  |  |  |  |
| *M1 segment* | 20 (24%)^A^ | 10 (13%)^D^ | 25 (31%)^B^ | 12 (16%)^D^ | 45 | 22 |
| *M2 segment* | 19 (25%)^D^ | 8 (11%)^D^ | 11 (15%)^D^ | 1 (1%)^D^ | 30 | 9 |
| Internal carotid artery |  |  |  |  |  |  |
| *(Supra-)clinoid segment* | 24 (29%)^A^ | 13 (17%)^D^ | 28 (34%)^A^ | 16 (21%)^D^ | 52 | 29 |
| *Terminal segment* | 21 (26%)^A^ | 6 (8%)^D^ | 22 (27%)^B^ | 11 (15%)^E^ | 43 | 17 |

***Online Supplemental Table 1.*** *The total number of (enhancing) vessel wall lesions, specified by arterial segment. Mean (in percentages) was calculated as total number of lesions that were detected in the specified segment over all patients divided by the number of segments that could be assessed over all patients. The number of patients in whom a segment could be assessed: ^A^ n=82, ^B^ n=81, ^C^ n=80, ^D^ n=76, ^E^ n=75.*

**Online Supplemental Table 2.** Overview of cerebral parenchymal changes, categorized by type.

| ***Type of infarct*** | *Left hemisphere* | *Right hemisphere* | *Anterior circulation* | *Posterior circulation* | *Total* |
| --- | --- | --- | --- | --- | --- |
| Cortical infarcts | 52 | 70 | 112 | 10 | 122 |
| Small subcortical infarcts | 0 | 1 | 1 | 0 | 1 |
| Lacunes of presumed vascular origin | 22 | 32 | 49 | 5 | 54 |
| Deep grey matter infarcts | 10 | 11 | 18 | 3 | 21 |
| Cortical microinfarcts | 20* | 23* | 40* | 3* | 43* |
|  |  |  |  |  |  |
| ***Type of white matter hyperintensities*** | *Score = 0* | *Score = 1* | *Score = 2* | *Score = 3* | *Total* |
| Periventricular white matter hyperintensities | 14 | 44 | 14 | 10 | 82 |
| Deep white matter hyperintensities | 14 | 43 | 16 | 9 | 82 |

***Online Supplemental Table 2.*** *The total number and type of infarct scored over all patients (n = 82), specified by hemisphere and anterior vs. posterior circulation. For all patients the grade of white matter hyperintensities was scored, specified in periventricular or deep white matter hyperintensities. Fazekas grade was described as: 0 = absence or single punctate white matter hyperintensity (deep), 1 = ‘caps’ or pencil-thin lining or multiple punctate lesions, 2 = smooth ‘halo’ or beginning confluency of lesions, 3 = large confluent lesions or irregular hyperintensities extending into the deep white matter (*[*28*](#_ENREF_28)*). * Due to motion artifacts, the number of scans that could be assessed was 79.*
